# A phase II study of single-agent gefitinib as first-line therapy in patients with stage IV non-small-cell lung cancer

**DOI:** 10.1038/sj.bjc.6603159

**Published:** 2006-05-02

**Authors:** R Suzuki, Y Hasegawa, K Baba, H Saka, H Saito, H Taniguchi, M Yamamoto, S Matsumoto, K Kato, T Oishi, K Imaizumi, K Shimokata

**Affiliations:** 1 Toyohashi Municipal Hospital, 50 Hachiken-nishi, Aotake, Toyohashi, Aichi 441-8570, Japan; 2 Nagoya University Graduate School of Medicine, 65 Tsuruma, Showa-ku, Nagoya 466-8550, Japan; 3 Aichi Medical University, 21 Karimata, Iyazako, Nagakute, Aichi 480-1195, Japan; 4 Nagoya Medical Center, 4-1-1, Sannomaru, Naka-ku, Nagoya 460-0001, Japan; 5 Aichi Cancer Center, 18 Kake-machi, Kuriyado, Okazaki, Aichi 444-0011, Japan; 6 Tosei General Hospital, 160 Nishioiwake, Seto, Aichi 489-8642, Japan; 7 Nagoya Ekisaikai Hospital, 4-66, Syonen-cho, Nakagawa-ku, Nagoya 454-8502, Japan; 8 Komaki Municipal Hospital, 1-20, Tsunebushin, Komaki, Aichi 485-8520, Japan; 9 Handa Municipal Hospital, 2-29, Toyo-cho, Handa, Aichi 475-8599, Japan

**Keywords:** gefitinib, IRESSA, non-small-cell lung cancer

## Abstract

The aim of this study was to evaluate the efficacy and tolerability of gefitinib (‘IRESSA’) in Japanese patients with previously untreated stage IV non-small-cell lung cancer (NSCLC). This was a multi-institutional phase II study. Thirty-four patients with previously untreated stage IV NSCLC were enrolled between May 2003 and September 2004. Gefitinib was administered orally 250 mg once a day and was continued until there was either disease progression or severe toxicity. Objective tumour response rate was 26.5% (95% confidence interval, 11.7–41.3%). Adverse events were generally mild (National Cancer Institute-Common Toxicity Criteria grade 1 or 2) and consisted mainly of skin rash, fatigue and liver dysfunction. No pulmonary toxicity was observed. The global health status revealed that there was no change in quality of life during the study. This study found that single-agent gefitinib is active and well tolerated in chemonaive Japanese patients with advanced NSCLC.

Gefitinib is an epidermal growth factor receptor (EGFR) tyrosine kinase (TK) inhibitor that blocks signal pathways involved in proliferation and survival of cancer cells (Wakeling *et al*, 1996). A phase I clinical trial showed that gefitinib is well tolerated and active in solid, malignant tumours (Ranson *et al*, 2002). Two large randomised phase II studies in patients with non-small-cell lung cancer (NSCLC) who had progressive disease (PD) following platinum-based chemotherapy, IRESSA Dose Evaluation in Advanced Lung Cancer (IDEAL 1) and 2 demonstrated response rates of 12–18%. (Fukuoka *et al*, 2003; Kris *et al*, 2003). A higher response rate of 28% was observed in Japanese patients in IDEAL 1, which is comparable to the response rate of 19% shown in the ECOG randomised study of four platinum-based chemotherapy regimens (Schiller *et al*, 2002). Gefitinib showed encouraging response rate (23%, 5 out of 22) and survival benefit (median survival 12.6 months) as a first-line therapy in NSCLC in the compassionate use (Argiris and Mittal, 2004) and a response rate of 27% (10 out of 37) in Japanese chemonaive patients with advanced NSCLC (Niho *et al*, 2004). The adverse effects of gefitinib were generally mild such as skin eruption, diarrhoea and nausea. These data suggest that further clinical evaluation of gefitinib in Japanese chemonaive patients is warranted. Therefore, we evaluated the efficacy and tolerability of gefitinib as a first-line therapy in a Japanese patient population. When disease progression was observed, the standard platinum-based doublet chemotherapy was considered to use as salvage.

## MATERIALS AND METHODS

### Patients

This study began in May 2003 and ended in September 2004. Patients inclusion criteria were as follows: (1) Stage IV NSCLC without previous chemotherapy or radiotherapy; (2) patients who had measurable lesions; (3) patients whose ages were 20⩽age<75; (4) patients whose Eastern Cooperative Oncology Group-Performance Status (ECOG-PS) were 0 or 1; (5) patients who had adequate organ function; partial pressure of oxygen in arterial blood (PaO_2_) ⩾70 Torr, adequate bone marrow (absolute neutrophil count ⩾1500 mm^−3^, platelets ⩾100 000 mm^−3^, haemoglobin ⩾10 g dl^−1^), renal function (serum creatinine ⩽1.25 × upper normal limit) and hepatic function (serum glutamic–oxaloacetic transaminase and serum glutamic pyruvic transaminase ⩽1.25 × upper normal limit). All patients with interstitial pneumonia were strictly excluded by chest computed tomography (CT).

All patients gave written informed consent before enrollment. This protocol was approved by the Institutional Review Boards of the participating centres.

### Treatment

Patients received gefitinib 250 mg orally, once a day. Study treatment was continued until disease progression or severe toxicity. In these cases, patients received carboplatin (CBDCA) and paclitaxel (TXL) chemotherapy as second-line therapy. Patients received TXL 200 mg m^−2^ as a 1-h intravenous infusion, followed by CBDCA area under the curve (AUC) 6.0 (Calvert's setting) as a 1-h infusion on day 1. Courses of treatment were repeated every 3 or 4 weeks depending on the recovery of toxicity.

Tumour response was assessed as complete response (CR), partial response (PR), stable disease *≧*12 weeks (SD), or PD in accordance with the standard Response Evaluation Criteria In Solid Tumours (RECIST). Toxicity was evaluated using National Cancer Institute-Common Toxicity Criteria (NCI-CTC) (version 2). Quality of life (QOL) was evaluated before the start of therapy and at 4 and 12 weeks, using the European Organisation for Research and Treatment of Cancer Care Quality of Life Questionnaire (EORTC-QLQ-C30) and lung-cancer-specific module (LC13) (Aaronson *et al*, 1993).

### Statistical considerations

The primary end point was response rate. The secondary end points were toxicity, survival from the date of enrolment and QOL. According to Simon's minimax two-stage phase II study design, the treatment programme was designed for a minimal response rate of 15% and to provide a significance level of 0.10 with a statistical power of 80% in assessing the activity of the regimen according to a 35% response rate. The upper limit for first-stage drug rejection was two responses in 17 evaluable patients. The upper limit for second-stage drug rejection was nine responses in 32 evaluable patients. We chose a sample number of 34, to allow for two dropouts. Tumour responses in subsets were compared using Fisher's exact test. Quality-of-life analyses were performed using Wilcoxon's signed-rank test. All *P*-values were considered significant if ⩽0.05.

## RESULTS

### Patient characteristics and treatment administration

Thirty-four patients were enrolled and all of them were eligible. Patient characteristics are shown in Table 1. Of the 34 patients, 21 were men and 13 were women. Median age was 64 years (range: 43–73). The ECOG-PS was 0 in 16 patients and 1 in 18 patients. The predominant histology was adenocarcinoma (25 patients). Twenty-three patients had smoking history (including current smoker or ex-smoker). Of the 34 patients, 33 patients discontinued gefitinib treatment. The reasons for discontinuing treatment were progression of disease (*n*=30), toxicities (skin; *n*=1, liver; *n*=1) and patient request (*n*=1). The median treatment period was 2.4 months (range: 1–23 months). Only one patient is still receiving treatment with gefitinib.

### Response

Nine patients (26.5%) achieved PR, and eight patients (23.5%) had SD as their best response. The objective response rate was 26.5% (95% CI, 11.7–41.3%). Subset analysis is shown in Table 2. Seven out of the 13 female patients achieved PR. There was a statistically significant difference between men and women. However, other factors such as pathology, smoking history and ECOG-PS demonstrated no significant difference. Median duration of response was 8.8 months (range 2.1–22.0 months). Median follow-up was 15.8 months (range 2.9–33.8 months).

### Overall survival

**Figure 1:**
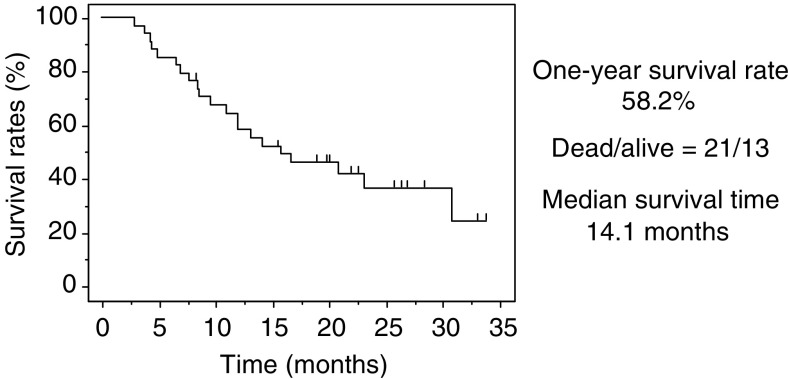
Kaplan–Meier survival curve.

Median survival time was 14.1 months. The 1-year survival rate was 58.2%. Twenty-one patients died during the study period, and 13 patients are still alive. The Kaplan–Meier survival curve is shown in Figure 1.

### Toxicities

Thirty-three patients were evaluated for toxicity. One patient demonstrated liver dysfunction from disease progression within 1 week of enrolling and was not evaluated for toxicity assessment. Generally, toxicities were mild. The most common toxicities were skin rash, general fatigue and liver dysfunction (Table 3). Most were grade 1 or 2 in severity and four patients (11.8%) experienced grade 3 toxicities (one skin rash, one general fatigue and four liver dysfunction). There was no pulmonary toxicity during the treatment.

### Quality of life

The global health status revealed no significant differences between QOL before gefitinib therapy and 4 or 12 weeks after commencing therapy, compared to baseline. Quality of life-30 scores were 4.7±1.7 before therapy, 4.9±1.3 after 4 weeks and 4.9±1.1 after 12 weeks (Table 4). For the other scales, at 12 weeks, improvement of insomnia and constipation, worsening of appetite loss, diarrhoea, financial difficulties and alopecia were observed (Tables 4 and 5).

### Response to subsequent chemotherapy

In total, 19 patients received second-line chemotherapy (CBDCA+TXL) subsequent to gefitinib. One had a CR, five had PR, nine had SD, and four had PD. Overall response rate was 31.6% (6 out of 19); 95% CI was 10.7–52.5%. The reasons of the 14 patients (one patients is still receiving gefitinib) who have not received the subsequent chemotherapy were patient's rejection to chemotherapy treatment (*n*=8), patient's request for other regimens (*n*=3) and worsening of general condition (*n*=3).

## DISCUSSION

Lung cancer is a major cause of cancer death in many countries in the world (Greenlee *et al*, 2000). Despite advances in treatment, a large meta-analysis of randomised trials demonstrated insufficient outcome of a 1-year survival benefit of 10% and a modest improvement in the median survival of 1.5 months for patients who were treated with platinum-containing regimens, compared with best supportive care (Non-small Cell Lung Cancer Collaborative Group, 1995). In addition, severe toxicities associated with platinum-based chemotherapy often led to treatment discontinuation. Therefore new drugs with novel modes of action that are effective and have less toxicities are needed. Gefitinib is a targeted drug with single-agent activity, and has shown mild toxicity in platina pretreated patients with NSCLC (Fukuoka *et al*, 2003; Kris *et al*, 2003).

As first-line therapy, gefitinib has been assessed in combination with two different chemotherapy regimens in two large randomised studies (IRESSA NSCLC Trial Assessing Combination Treatment (INTACT) 1 and INTACT 2) (Giaccone *et al*, 2004; Herbst *et al*, 2004). Disappointingly, both studies failed to show an improvement in either survival or other clinical end points.

A large placebo-controlled phase III study in patients with advanced NSCLC who had received one or two prior chemotherapy regimens, IRESSA Survival Evaluation in Lung Cancer (ISEL), showed some improvement in overall survival with single-agent gefitinib that failed to reach statistical significance compared with placebo in the overall or adenocarcinoma co-primary populations. However, there was marked heterogeneity in survival outcomes between patient groups, with some evidence of benefit among never-smokers and patients of Asian origin (Thatcher *et al*, 2005).

Little information is available for single-agent gefitinib as first-line therapy, and the available data are from compassionate use of gefitinib with a small number of patients (Argiris and Mittal, 2004; Niho *et al*, 2004).

In the present study, the overall response rate was 26.5%. We demonstrated that gefitinib was active in patients with NSCLC as first-line single-agent therapy. It has been reported that gefitinib is more effective in women, in nonsmokers, in patients affected with adenocarcinoma and in patients of East Asian ethnicity (Fukuoka *et al*, 2003; Kris *et al*, 2003; Miller *et al*, 2004; Ho *et al*, 2005). As all of our patients were Japanese, it is not surprising that this response rate is higher than would be expected in other ethnicities (Argiris and Mittal, 2004; Kommareddy *et al*, 2004). In our subset analysis, women had a higher response rate than men (53.8 *vs* 9.5%). However, other predicting factors such as smoking history or histology did not show any significant differences, possibly due to the small sample size.

Mutations in the TK domain of the EGFR have been identified in some patients with refractory NSCLC who achieved good responses to gefitinib (Cappuzzo *et al*, 2004; Lynch *et al*, 2004; Pao *et al*, 2004). Further recent studies suggest that the mutation status of EGFR-TK may predict clinical benefits with gefitinib (Marchetti *et al*, 2005; Mitsudomi *et al*, 2005; Yang *et al*, 2005). Different efficacy in different ethnicities may be explained by the different frequency of these mutations in different ethnic groups.

Quality of life and toxicity are major considerations in the therapy of advanced lung cancer. It was reported that an improvement in QOL was observed in a gefitinib compassionate-use programme (Mu *et al*, 2004). In the present study, global health status did not change during gefitinib therapy. At 12 weeks of gefitinib therapy, improvement of insomnia and constipation, but worsening of appetite loss, diarrhoea, financial difficulties and alopecia were observed. Treatment with single-agent gefitinib therapy did not result in worsening of symptoms such as fatigue, nausea and vomiting.

Recently, concerns have arisen about an infrequent but serious side effect of gefitinib – interstitial pneumonia. A large randomised phase II study (IDEAL 1; Fukuoka *et al*, 2003) reported only two interstitial pneumonia cases out of 210 patients owing to gefitinib, and these cases were at a 500-mg dose of gefitinib. However, a surprisingly high incident rate of interstitial pneumonia (four of 18 patients) was reported from Japan (Inoue *et al*, 2003). Indeed, in a phase II study from Japan, four patients out of 42 patients developed grade 5 interstitial pneumonia (Niho *et al*, 2004). The incidence of pulmonary toxicity is thought to be higher in Japanese than in other ethnicities (FDA, 2003). Furthermore, it has been reported that the incidence of interstitial lung disease (ILD) in the Japanese patients on gefitinib was 4.5%, and it was higher in the patients with pre-existing pulmonary fibrosis (Hotta *et al*, 2005). Furthermore, many chemotherapeutic agents have ILD information and precautions documented within their Japanese prescribing information. Reasons for the difference in reporting rate between Japan and the rest of world are unknown and require further scientific investigation. In the present study, we strictly excluded patients who had pulmonary fibrosis by chest CT, and no pulmonary toxicity occurred. Thus, in East Asia, patients should be very carefully selected and observed during gefitinib therapy.

In our study, the median survival time was 14.1 months, and the 1-year survival rate was 58.2%. Common toxicities were skin rash, general fatigue and liver dysfunction. However, there was no significant haematological or pulmonary toxicity. Gefitinib showed antitumour activity, even in stage IV NSCLC, with a good survival outcome.

In conclusion, phase III studies are warranted to compare platinum-containing regimens *vs* gefitinib alone in the East Asian population.

## Figures and Tables

**Table 1:** Patient characteristics

**Sex**	**Male/female**	**21/13**
Age	Median (range)	64 (43–73)
ECOG-PS	0/1	16/18
Histology	Ad/Sq/Other	25/5/4
Smoking history	Yes/never	23/11
Clinical stage	Stage IV	34

Ad=adenocarcinoma; Sq=squamous-cell carcinoma; ECOG-PS=Eastern Cooperative Oncology Group Performance Status.

**Table 2:** Subset analysis (response rate)

**Patient characteristics**		**Response rate**	***P*-value**
Sex	Male *vs* female	2/21 (9.5%) *vs* 7/13 (53.8%)	0.0057
Pathology	Ad *vs* Sq+other	7/25 (28%) *vs* 2/9 (22.2%)	0.7432
Smoking	Current+ex-smoker *vs* never smoker	4/23 (17.4%) *vs* 5/11 (45.5%)	0.1009
ECOG-PS	0 *vs* 1	5/16 (31.3%) *vs* 4/18 (22.2%)	0.5633

Ad=adenocarcinoma; Sq=squamous-cell carcinoma; ECOG-PS=Eastern Cooperative Oncology Group Performance Status.

**Table 3:** Toxicities (NCI-CTC version 2.0)

	**No. of patients**			
**Grade**	**1**	**2**	**3**	**Total**
Skin rash	14	5	1	20
General fatigue	6	3	1	10
Liver dysfunction	2	4	4	10
Diarrhoea	6	0	0	6
Nausea	4	2	0	6
Anaemia	0	1	0	1

NCI-CTC=National Cancer Institute-Common Toxicity Criteria.

There were no pulmonary toxicities.

**Table 4:** Evaluation of the quality of life of patients using the QLQ-C30 questionnaire

	**Before therapy *n*=34**	**After 4 weeks *n*=32**	**After 12 weeks *n*=20**
Global health status/QOL	4.7±1.7	4.9±1.3	4.9±1.1
*Functional scales*			
Physical functioning	94.0±7.2	94.6±7.0	93.3±6.4
Role functioning	94.6±13.2	96.2±7.1	93.1±8.6
Emotional functioning	82.8±19.7	86.6±13.6	86.8±13.5
Cognitive functioning	85.5±15.4	86.6±14.5	87.5±14.4
Social functioning	73.7±26.8	79.6±21.4	81.9±23.0
Role functioning	94.6±13.2	96.2±7.1	93.1±8.6
			
*Symptom scales/items*			
Fatigue	19.4±17.0	23.7±21.0	19.4±14.3
Nausea and vomiting	0.5±3.0	1.6±5.0	2.8±6.5
Pain	18.3±17.4	14.5±13.4	15.3±16.6
Dyspnoea	15.1±18.9	19.4±24.0	13.9±17.2
Insomnia	30.1±30.3*	14.0±18.8*	11.1±16.4*
Appetitie loss	9.7±15.4	18.3±24.0*	22.2±29.6*
Constipation	14.0±18.8	15.1±22.5	0^*^
Diarrhoea	5.4±12.5	12.9±18.6	25.0±32.2*
Financial difficulties	21.5±31.7	21.5±28.0	30.6±38.8*

QLQ-C30=Quality of Life Questionnaire; QOL=quality of life.

* *P*<0.05 compared with before therapy.

**Table 5:** QOL evaluation (LC13)

	**Before therapy *n*=34**	**After 4 weeks *n*=32**	**After 12 weeks *n*=20**
*Symptom scales/items*			
Dyspnoea	12.5±17.1	14.0±17.7	11.1±16.4
Coughing	32.3±15.8	24.7±21.0	22.2±16.4
Haemoptysis	4.2±11.2	8.6±17.1	8.3±15.1
Sore mouth	6.3±9.9	9.7±17.6	5.6±13.0
Dysphagia	3.1±9.9	6.5±15.9	11.1±16.4
Peripheral neuropathy	15.6±17.2	14.0±18.8	13.9±17.2
Alopecia	3.1±13.0	7.5±18.7	16.7±17.4*
Chest pain	10.4±15.7	7.5±14.2	13.9±17.2
Arm or shoulder pain	7.3±18.4	10.8±18.0	19.4±33.2
Pain in other regions	26.9±27.8	18.3±20.8	16.7±17.4

QOL=quality of life; LC13=lung-cancer-specific module.

* *P*<0.05 compared with before therapy.
